# Associations between Rheumatoid Arthritis and Various Comorbid Conditions in Germany—A Retrospective Cohort Study

**DOI:** 10.3390/jcm12237265

**Published:** 2023-11-23

**Authors:** Candice Aphroditta Imanuel, Sathiha Sivatheesan, Ai Koyanagi, Lee Smith, Marcel Konrad, Karel Kostev

**Affiliations:** 1 Epidemiology, IQVIA, Unterschweinstiege 2–14, 60549 Frankfurt am Main, Germany; 2 Research and Development Unit, Parc Sanitari Sant Joan de Déu, Sant Boi de Llobregat, 08830 Barcelona, Spain; 3 Centre for Health Performance and Wellbeing, Anglia Ruskin University, Cambridge CB1 1PT, UK; 4 Health & Social, FOM University of Applied Sciences for Economics and Management, 60486 Frankfurt am Main, Germany; 5 University Hospital, Philipps University Marburg, 35043 Marburg, Germany

**Keywords:** rheumatoid arthritis, comorbidity, co-diagnosis, general practice, primary care

## Abstract

Aims: The aim of the present study was to estimate the prevalence of physical and mental health comorbidities in patients with rheumatoid arthritis (RA) in Germany, in order to better understand the complex clinical picture and its consequences. Methods: This retrospective cohort study was based on data from the IQVIA Disease Analyzer database and included individuals aged ≥ 16 years with an initial documented diagnosis of RA between 2015 and 2021 (index date). RA patients were matched 1:1 with non-RA individuals using nearest neighbor propensity score matching. The study investigated associations between RA and various disorders documented within 365 days after the index date. The cumulative incidence of defined disorders was calculated for individuals with and without RA. Multivariable logistic regression models were used to study these associations. Results: Data were available for a total of 49,713 patients with and 49,713 patients without RA (mean age: 60.4 (SD: 15.5) years; 66.7% female). A significant and clinically relevant positive association was observed between RA and seven disorders: soft tissue disorders (Odds Ratio (OR): 1.47; 95% Confidence Interval (CI): 1.42–1.52), renal failure (OR: 1.36; 95% CI: 1.26–1.47), anemia (OR: 1.32; 95% CI: 1.24–1.40), liver diseases (OR: 1.32, 95% CI: 1.23–1.41), osteopathies and chondropathies (OR: 1.28; 95% CI: 1.22–1.3), diseases of the esophagus, stomach and duodenum (OR: 1.18; 95% CI: 1.14–1.22), and nutritional deficiencies (OR: 1.17; 95% CI: 1.10–1.24). Conclusions: We observed significant associations between RA and several comorbidities, which have clinical relevance for the care of RA patients not only in general practices but also in specialist settings.

## 1. Introduction

Rheumatoid arthritis (RA) is a chronic, inflammatory, systemic autoimmune disease that affects approximately 1% of the population worldwide [1,2]. One study reported that the average prevalence of RA in the German population was 1.26% over six years (2008–2013) [3]. Generally, the likelihood of women developing RA is 2–3 times higher than that of men. The estimated cumulative lifetime risk of developing adult-onset RA is approximately 3.6% for women and 1.7% for men [4,5]. RA is associated with progressive disability and systemic complications. It imposes a significant burden on society and causes high morbidity [1,2,6]. The disease is characterized by inflammation and overgrowth of the synovium, the presence of autoantibodies such as rheumatoid factor (RF) and anti-citrullinated protein antibodies (ACPA), deformities in cartilage and bone, and various manifestations, including cardiovascular, pulmonary, psychological, skin, and skeletal disorders [6]. It causes a reduction in quality of life (QoL), impacting the physical, emotional, economic, and occupational aspects of patients’ lives [7]. Over the years, the treatment of patients with RA has improved [8]. The current guidelines for the management of early arthritis established by The European Alliance of Associations for Rheumatology (EULAR) endorse prompt specialist referral, emphasizing the achievement of a reduction of at least 50% in clinical disease activity within 3 months of treatment initiation, with the ultimate treatment objective being the attainment of remission within a 6-month timeframe [9]. The achievement of these ambitious objectives has been facilitated by the expanded utilization of methotrexate and the introduction of TNF-α inhibitors since the beginning of the 21st century [10]. Other revolutionary treatment options for rheumatoid arthritis include abatacept (Orencia), which is a biological drug that blocks the activation of T cells. Another is tocilizumab (Actemra). This is a biological drug that blocks IL-6, a chemical messenger of inflammation. Finally, there is tofacitinib (Xeljanz). These are also known as JAK inhibitors, as they use a different mechanism of action. JAK inhibitors block the Janus kinases in the cells, which are enzymes of inflammation [11].

Over the past decade, there has been a growing focus on comorbidities among patients with rheumatic diseases in both research and clinical care. It is important to note that both pharmacological therapy for and surgical management of patients with rheumatic conditions are affected by comorbidities. The occurrence of comorbidities is linked to adverse health consequences, which encompass diminished functionality, decreased quality of life, and heightened morbidity and mortality rates [12]. Comorbidities are widely considered a significant concern among patients with RA, especially since comorbidity poses a potential risk to the long-term prognosis and overall improvement in patients with the condition [13]. Often, patients with RA have two or more comorbid conditions [14]. Currently, there is a limited number of published studies that provide quantitative data on the prevalence of comorbidities in individuals with RA. However, the majority of published papers consist of case reports or clinical/hospital studies that focus on either a single autoimmune disease or a selection of several autoimmune diseases [15,16,17,18,19]. For example, a study in the US on the prevalence of co-existing autoimmune diseases in RA patients found that patients with RA were slightly more likely to have chronic obstructive pulmonary disease (COPD) [13]. The Comorbidities in Rheumatoid Arthritis (COMORA) study, a comprehensive international cross-sectional study involving 3920 RA patients from 17 different countries, examined the prevalence of comorbidities. Among the most frequently reported comorbidities, whether past or present, were depression (15%), asthma (7%), cardiovascular events such as myocardial infarction (MI) and stroke (6%), solid-organ malignancies (5%), and chronic obstructive pulmonary disease (4%). The COMORA study revealed significant variations in the prevalence of these comorbidities between countries. For instance, the prevalence of depression ranged from 2% in Morocco to 33% in the USA. In RA patients, comorbidities like cardiovascular disease (CVD), infections, and malignancies are significant, as they can increase the risk of mortality [20].

It is essential that we gain a better understanding of the epidemiology of multimorbidity in order to facilitate the development of interventions aimed at prevention, burden reduction, and the precise alignment of healthcare services with individual patient needs. The aim of this study is therefore to identify the correlation between rheumatoid arthritis and various physical and mental health comorbidities in patients in Germany in order to better understand the complex clinical picture and its consequences.

## 2. Methods

### 2.1. Database

The present study was based on data from the IQVIA Disease Analyzer database. This data source consists of electronic medical records (baseline demographical data, prescriptions, and diagnoses) obtained directly and in an anonymous format from computer systems used by office-based general practitioners and specialists [21]. The database contains approximately 3000 physicians who were initially selected using a panel design that was based on the following strata: specialist group, German federal state, community size category, and physician age. Previous research has shown that the database is representative of the overall situation in the country [21]. It has previously been used in many studies on epidemiology, including patients with RA [22,23].

### 2.2. Study Population

This retrospective cohort study included individuals aged 16 years or older with an initial documented diagnosis of RA (ICD-10: M05, M06) between January 2015 and December 2021 (index date). Only patients with an observation time of at least 12 months prior to the index date and a follow-up time of least 365 days after the index date were included. This inclusion criterion was necessary to estimate the incidence of RA and facilitate the analysis of initial diagnoses documented within 12 months following RA diagnosis.

**Figure 1:**
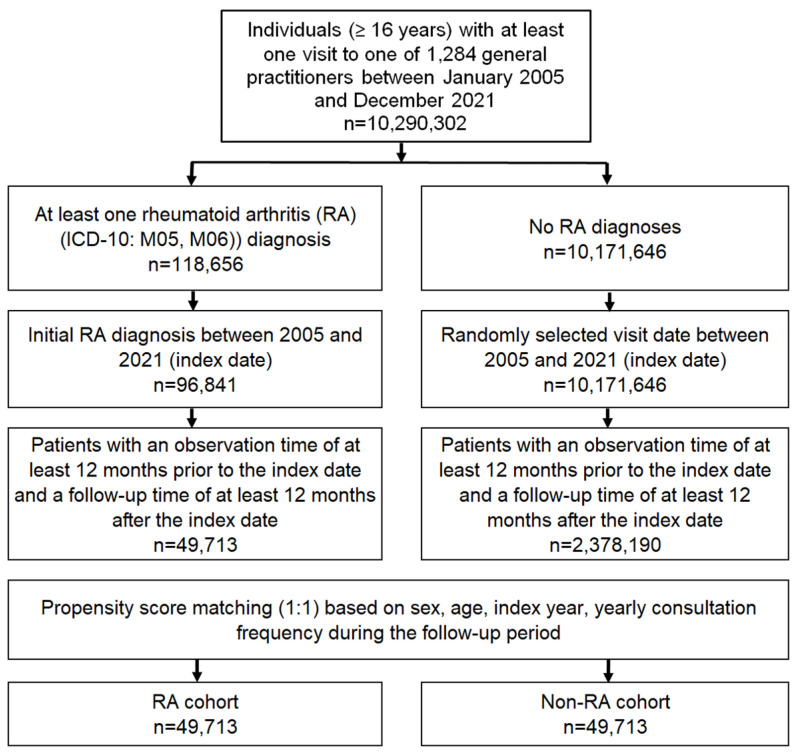
Selection of study patients.

RA patients were matched 1:1 with non-RA individuals using nearest neighbor propensity score matching based on age, sex, index year (year of index date), and consultation frequency during the follow-up period. The index date for the non-RA individuals was that of a randomly selected visit to the physician between January 2015 and December 2021 (Figure 1).

### 2.3. Study Outcomes and Covariates

The study outcomes were the associations between RA and various physical and mental disorders documented within 365 days after the index date. All diagnoses or diagnosis classes that occurred in at least 3% of the study patients were analyzed. These disorders included neoplasms; diseases of the blood and blood-forming organs; endocrine, nutritional, and metabolic diseases; diseases of the nervous, circulatory, respiratory, digestive, musculoskeletal, and urinary systems; fractures; and mental disorders.

### 2.4. Statistical Analyses

The cumulative incidence of defined disorders was calculated for individuals with and without RA. Multivariable logistic regression models with all study disorders as dependent variables and RA as the independent variable were used. To counteract the problem of multiple comparisons, *p*-values < 0.001 were considered statistically significant. Due to the large samples used, highly significant differences can be identified based on small absolute differences. To avoid this problem, results were considered clinically relevant when an OR was greater than 1.15 or less than 0.85. For clinically relevant results, regression analyses were repeated separately for female and male patients. Analyses were conducted using SAS version 9.4 (SAS Institute, Cary, NC, USA).

## 3. Results

### 3.1. Basic Characteristics of the Study Sample

After 1:1 matching, the present study included 49,713 patients with and 49,713 patients without RA. The basic characteristics of the study patients are listed in Table 1. The mean age (SD) was 60.4 (15.5) years; 66.7% were female.

### 3.2. Association between RA and Pre-Defined Physical and Mental Disorders

The prevalence of pre-defined chronic disorders is shown in Table 2. A significant and clinically relevant positive association was observed between RA and seven disorders: soft tissue disorders (ICD-10: M60–M79) (56.4% vs. 44.3%; Odds Ratio (OR): 1.47; 95% Confidence Interval (CI): 1.42–1.52), renal failure (ICD-10: N17–N19) (8.5% vs. 6.6%; OR: 1.36; 95% CI: 1.26–1.47), anemias (ICD-10: D50–D64); 18.2% vs. 13.9%; OR: 1.32; 95% CI: 1.24–1.40), liver diseases (ICD-10: K70–K77) (15.6% vs. 13.2%; OR: 1.32, 95% CI: 1.23–1.41), osteopathies and chondropathies (ICD-10: M80–M94) (31.0% vs. 25.0%; OR: 1.28; 95% CI: 1.22–1.3), diseases of the esophagus, stomach, and duodenum (ICD-10: K20–K31) (43.7% vs. 36.9%; OR: 1.18; 95% CI: 1.14–1.22), and nutritional deficiencies (ICD-10: E40–E64) (14.3% vs. 11.1%; OR: 1.17; 95% CI: 1.10–1.24) (Table 2). The Odds Ratios were similar in female and male patients (Table 3).

## 4. Discussion

In the present retrospective study including 49,713 patients, a notable association was observed between RA and several comorbidities, including soft tissue disorders; renal failure; anemias; liver diseases; osteopathies and chondropathies; diseases of the esophagus, stomach, and duodenum; and nutritional deficiencies. In comparison with previous similar studies, the association and the prevalence between RA and the comorbidities found in our study are similar.

This study found an association between RA and various soft tissue disorders. A review focusing on the association between RA and myositis showed various studies supporting this association [24]. RA is found in 30% of idiopathic inflammatory myopathy patients [24]. Another study also confirmed the association between RA and other joint inflammation [25]. Shoulder lesion is commonly reported in RA patients; overall, 48% of RA patients develop erosive changes within the shoulder, which results in pain and impacts shoulder function [26,27]. The underlying mechanism behind this association could be systemic inflammation and the associated elevated cytokine levels [28,29].

In our study, RA was associated with an increase in the prevalence of renal failure. In previous studies, the documented prevalence of renal failure in RA patients varied between 5% and 50% depending on the diagnostic criteria, definition of renal disease, and study design [30,31,32]. A national cohort study from China found that the risk of renal diseases (chronic kidney disease, glomerulonephritis, and end-stage renal disease) is significantly higher in RA patients [33]. The development of renal disease in RA patients is influenced by various factors and processes, such as chronic inflammation, comorbidities, nephrotoxic antirheumatic drugs, and renal involvement associated with RA [31,32,34]. A further finding of our study, which is in line with published research, is the increased prevalence of anemia in RA patients. Anemia is a common comorbidity among patients with RA, occurring in 15% to 47% of patients with the condition [35,36,37]. The two main proinflammatory cytokines in RA, namely IL-6 and TNFa, are crucial factors that can induce inflammatory anemia via the synthesis of hepcidin, which reduces intestinal iron absorption and prevents iron release [38]. TNFa is associated with alterations in the process of erythropoiesis [38]. Chen at al. found that Hb levels were significantly lower in RA patients than in the general population [35]. Anemia prevalence was significantly higher in RA patients (47%) than in the control group (4.4%). Chen et al. also compared non-anemic and anemic RA patients and found that anemia status was significantly related to higher levels of disease activity, greater structural damage, and worse joint function [35]. In their study, the Hb level was found to be a protective factor for disease activity and structural damage in RA patients [35].

The association between RA and osteopathies or chondropathies is a further finding of our study. The risk of osteopathies and chondropathies is increased in patients with RA, and especially in women (OR: 1.31 (1.25–1.38) in women vs. 1.18 (1.08–1.29) in men). This finding is in line with a similar study in Germany, which found that osteoarthritis (44%) and osteoporosis (25.9%) were the most prevalent comorbidities in people with RA [39]. Kareem et al. assessed the risk factors for osteoporosis development in patients with RA and categorized the factors into three groups: factors related to the patient, factors related to RA, and those related to treatment [40]. Female and elderly patients with low levels of vitamin D, calcium and omega 3, a genetic predisposition to osteoporosis, and a family history of osteoporosis have a higher risk of osteoporosis [40]. The chronic inflammation, calcium malabsorption, and immobility associated with RA and its treatment, involving the application of glucocorticoids over a long duration, increase the risk of developing osteoporosis in individuals with RA [40].

This study supports previous findings indicating an association between RA and liver disease. Indeed, between 18% and 50% of RA patients have been reported as having abnormal liver test results, and 65% of RA patients have abnormal liver biopsy findings [41]. With regard to liver damage in RA patients, the main cause could either be the hepatic manifestation of RA, which is associated with primary liver disease, or hepatotoxic liver disease due to RA treatment [42]. The first stage of liver damage in RA commonly begins with asymptomatic abnormal liver tests, which may then develop into cirrhosis [42]. Liver damage is a commonly reported adverse event in RA patients receiving nonsteroidal anti-inflammatory drugs (NSAIDs) and methotrexate (MTX) therapy [43,44].

The results of our study indicate an association between RA and various diseases of the gastrointestinal system (esophagus, stomach, and duodenum). Previous studies have documented similar findings. Esophageal motility problems are commonly found in RA patients, resulting in dysphagia in 13% to 33.3% of patients [45]. Esophageal disease in RA patients may be caused by inflammation, therapy side effects or both [46]. The typical RA-related diseases that manifest in the stomach include nausea, vomiting, gastrointestinal bleeding, and dysmotility from amyloidosis [46].

Our study found a significant manifestation of nutritional deficiency in patients with RA. The results of previous studies also support this finding [47,48]. In a study focusing on older RA patients, Cano-Garcia et al. found that one-third of older RA patients have impaired nutritional status [48]. RA patients are at risk of nutritional deficiency due to fatigue, pain, loss of motor function, and nausea, which could result in the reduction of food consumption and consequently the decreased intake of vitamins and minerals [49]. MTX therapy is also associated with folic acid deficiency, and prolonged therapy with MTX is linked with chronic gastritis or peptic ulceration, both of which facilitate malabsorption [50,51,52].

Numerous studies have reported an association between RA and depression [53,54,55]. One similar study in Germany reported the prevalence of depression in RA patients as 32% [39], while another study with a different age sample and definition of depression reported a prevalence of 15% to 39% [56]. This difference could be caused by the difference in observation period. The main contributing factor for depression in patients with RA is the duration of suffering [57], and the one-year observation period may be too short to observe the development of depression. Another possible explanation could be the 1:1 matching of RA and non-RA patients on visit frequency.

Drug adverse event as the possible cause for comorbidities have been mentioned in this discussion. A retrospective study assessing RA patients reported a high prevalence of drug-related problems and multiple comorbidities being a factor that had significant association with drug-related problems [57]. Thus, the possibility that the occurrence of comorbidities as adverse effects of anti-rheumatic drugs could not be completely ruled out.

For individuals with RA, the findings of this study reveal a heightened prevalence of comorbidities including renal failure, anemia, osteopathies, chondropathies, liver diseases, gastrointestinal issues, and nutritional deficiencies. This result emphasizes the systemic nature of RA, highlighting the importance of taking a holistic approach to the treatment of RA patients including monitoring and screening, as well as intervention to manage specific comorbidities.

For healthcare professionals, this study illuminates the network of comorbidities associated with RA, stressing the need for a comprehensive management strategy. The heightened risk of renal failure underscores the necessity for vigilant monitoring of kidney function in RA patients. Addressing anemia becomes crucial not only for managing disease activity but also for improving joint outcomes. The link with osteopathies and chondropathies accentuates the need for targeted interventions to mitigate musculoskeletal complications. The awareness of potential liver damage and gastrointestinal manifestations requires the careful consideration of RA treatments and their impact. The increased prevalence of nutritional deficiencies adds an additional layer of concern, prompting physicians to address factors influencing dietary intake and absorption. A holistic and proactive approach, integrating both rheumatological and systemic care, is essential in order to optimize the health outcomes of individuals with RA.

### Strengths and Limitations

One strength of this study is its data source and the related large sample size. The study draws its data from the IQVIA Disease Analyzer database, a comprehensive and large source of electronic medical records encompassing baseline demographic data, prescriptions, and diagnoses from 3000 physicians, offering a substantial sample size. This can improve the statistical power of the analysis and enhance the generalizability of the findings. The retrospective cohort study design, including a minimum observation time of 12 months prior to the index date and a follow-up time of at least 365 days afterwards, allows for the examination of trends and associations over time. This design is particularly useful for studying chronic conditions like RA. The 1:1 propensity score matching of RA patients with a non-RA population helps control for potential confounders and thus enhances comparability between the two groups. To date, this is the first study to assess the association between RA and a wide range of physical and mental disorders, providing a comprehensive view on the impact of RA on various health outcomes and its clinical relevance. While the study boasts strengths such as a robust data source, large sample size, and comprehensive outcome analysis, it is not without limitations. The findings may not be entirely generalizable to the populations outside the scope of the physicians included in the Disease Analyzer Databank. As an observational study, this study may establish associations but not causation. Although this study used propensity score matching, it might still be subject to selection bias. There could still be unobserved factors that might influence RA and the related outcome, leading to selection bias. A further limitation is the lack of information on smoking status, alcohol use, and other lifestyle factors in the database. Finally, data from GPs were used to estimate different co-morbidities. Since rheumatologists primarily treat RA patients, they usually do not document non-rheumatological diagnoses. As data from rheumatologists could not be used for this study, we could not investigate the association between anti-RA therapy and subsequent comorbidities. A further limitation would be due to the change in the EULAR RA in 2010. Although the 1987 and 2010 diagnostic criteria have identical sensitivity, the latest EULAR RA diagnostic criteria have been proven to have higher specificity and performed better than its predecessor [58]. A few studies mentioned in the discussion were published before 2010 and had therefore diagnosed RA based on the 1987 criteria, which could be one limitation of this study.

## 5. Conclusions

In this study, we observed significant associations between RA and several comorbidities, which have clinical relevance with regard to the care of RA patients in general practices but also in specialist settings.

## Figures and Tables

**Table 1:** Baseline characteristics of study patients prior to and after propensity score matching.

	Prior to Matching			After Matching		
Variable	Proportion among Individuals with RA (*n*, %)	Proportion among Individuals without RA (*n*, %)	*p*-Value	Proportion among Individuals with RA (*n*, %)	Proportion among Individuals without RA (*n*, %)	*p*-Value
*n*	49,713	2,378,190		49,713	49,713	
Sex: female	33,142 (66.7)	1,255,094 (52.8)	<0.001	33,142 (66.7)	33,142 (66.7)	1.000
Sex: male	16,571 (33.3)	1,123,096 (47.2)		16,571 (33.3)	16,571 (33.3)	
Age (mean, SD)	60.4 (15.5)	52.2 (19.8)	<0.001	60.4 (15.5)	60.4 (15.5)	1.000
Age ≤ 50	12,392 (24.9)	1,078,807 (45.4)	<0.001	12,392 (24.9)	12,392 (24.9)	1.000
Age 51–60	12,098 (24.3)	433,568 (18.2)		12,098 (24.3)	12,098 (24.3)	
Age 61–70	10,579 (21.3)	364,163 (15.3)		10,579 (21.3)	10,579 (21.3)	
Age > 70	14,644 (29.5)	501,652 (21.1)		14,644 (29.5)	14,644 (29.5)	
Index year 2005–2008	5748 (11.6)	195,523 (8.2)	<0.001	5748 (11.6)	5748 (11.6)	1.000
Index year 2009–2012	9218 (18.5)	303,251 (12.8)		9218 (18.5)	9218 (18.5)	
Index year 2013–2016	15,075 (30.3)	532,896 (22.4)		15,075 (30.3)	15,075 (30.3)	
Index year 2017–2021	19,672 (39.6)	1,346,520 (56.6)		19,672 (39.6)	19,672 (39.6)	
Consultation frequency within 12 months after the index date (mean, SD)	8.2 (4.3)	5.9 (4.3)	<0.001	8.2 (4.3)	8.2 (4.3)	1.000

**Table 2:** Cumulative incidence of different disorders and association between RA and several disorders in individuals followed in general practices in Germany.

Diagnosis (ICD-10 Codes)	Proportion among Individuals with RA (%)	Proportion among Individuals without RA (%)	Odds Ratio for RA Patients (95% CI) *	*p*-Value
Clinically relevant associations				
Soft tissue disorders (M60–M79)	56.4	44.3	1.47 (1.42–1.52)	<0.001
Renal failure (N17–N19)	8.5	6.6	1.36 (1.26–1.47)	<0.001
Anemias (D50–D64)	18.2	13.9	1.32 (1.24–1.40)	<0.001
Diseases of liver (K70–K77)	15.6	13.2	1.32 (1.23–1.41)	<0.001
Osteopathies and chondropathies (M80–M94)	31.0	25.0	1.28 (1.22–1.33)	<0.001
Diseases of esophagus, stomach, and duodenum (K20–K31)	43.7	36.9	1.18 (1.14–1.22)	<0.001
Nutritional deficiencies (E40–E64)	14.3	11.1	1.17 (1.10–1.24)	<0.001
Significant but not clinically relevant associations				
Disorders of purine and pyrimidine metabolism (E79)	13.6	11.8	1.13 (1.09–1.18)	<0.001
Diabetes mellitus (E10–E14)	24.3	22.5	1.11 (1.08–1.15)	<0.001
Heart failure (I50)	10.4	9.1	1.11 (1.06–1.17)	<0.001
Ischemic heart diseases (I20–25)	21.9	19.6	1.10 (1.06–1.14)	<0.001
Extrapyramidal and movement disorders (G20–G26)	6.2	4.1	1.10 (0.99–1.21)	0.075
Chronic lower respiratory diseases (J40–J47)	40.0	36.3	1.07 (1.04–1.10)	<0.001
Dorsopathies (M40–M54)	74.3	69.5	1.07 (1.04–1.10)	<0.001
Thyroid gland disorders (E00–E07)	34.8	32.2	1.06 (1.03–1.09)	<0.001
Other diseases of kidney and urinary system (N20–N39)	31.4	30.5	0.92 (0.89–0.95)	<0.001
Fractures (S02, S12, S22, S32, S42, S52, S62, S72, S82, S92, T02, T08, T10, and T12)	12.4	12.1	0.92 (0.88–0.95)	<0.001
Cerebrovascular diseases (I60–I69)	9.9	10.1	0.92 (0.88–0.96)	<0.001
Sleep disorders (G47)	18.7	18.1	0.91 (0.88–0.95)	<0.001
Anxiety disorder (F41)	10.5	10.2	0.91 (80.87–0.95)	<0.001
No significant associations				
Somatoform disorders (F45)	21.9	19.0	1.06 (1.02–1.09)	0.002
Depression (F32, F33)	3.2	2.8	1.05 (1.02–1.08)	0.004
Hypertension (I10)	56.4	54.9	1.01 (0.98–1.04)	0.502
Diseases of veins, lymphatic vessels, and lymph nodes (I80–I89)	26.8	25.0	1.01 (0.97–1.04)	0.770
Reaction to severe stress and adjustment disorders (F43)	15.2	14.3	0.97 (0.93–1.00)	0.066
Cardiac arrhythmias (I46–I49)	17.5	16.4	0.99 (0.96–1.03)	0.712
Disorders of lipoprotein metabolism (E78)	38.2	37.1	0.96 (0.93–0.99)	0.005
Benign neoplasms (D10–D36)	14.4	13.7	0.95 (0.92–0.99)	0.010
Cancer (C00–C99)	11.7	11.7	0.94 (0.90–0.97)	0.001
Dementia (F01–F03, G30)	3.9	3.9	0.93 (0.87–1.00)	0.042
Obesity (E66)	14.2	15.6	0.91 (0.85–0.97)	0.005

* Multivariate logistic regression adjusted for all diagnoses listed in the table; *p* < 0.001 is considered statistically significant.

**Table 3:** Association between RA and defined disorders in individuals followed in general practices in Germany by sex.

	Women		Men	
Diagnosis (ICD-10 Codes)	Odds Ratio for RA (95% CI)	*p*-Value	Odds Ratio for RA (95% CI)	*p*-Value
Soft tissue disorders (M60–M79)	1.49 (1.43–1.55)	<0.001	1.44 (1.35–1.53)	<0.001
Renal failure (N17–N19)	1.41 (1.28–1.56)	<0.001	1.30 (1.14–1.47)	<0.001
Anemias (D50–D64)	1.30 (1.22–1.40)	<0.001	1.36 (1.22–1.51)	<0.001
Diseases of liver (K70–K77)	1.34 (1.23–1.47)	<0.001	1.26 (1.13–1.42)	<0.001
Osteopathies and chondropathies (M80–M94)	1.31 (1.25–1.38)	<0.001	1.18 (1.08–1.29)	<0.001
Diseases of esophagus, stomach, and duodenum (K20–K31)	1.18 (1.13–1.24)	<0.001	1.18 (1.11–1.25)	<0.001
Nutritional deficiencies (E40–E64)	1.18 (1.10–1.26)	<0.001	1.15 (1.02–1.29)	0.021

## Data Availability

The datasets used and analyzed during the current study are available from the corresponding author on reasonable request.
